# Key Role of Left Atrial Appendage during Redo Ablation in a Case of Long-Standing Persistent Atrial Fibrillation

**DOI:** 10.1155/2020/9691584

**Published:** 2020-06-19

**Authors:** Shaohui Wu, Guangchen Zou, Xu Liu, Weifeng Jiang, Mu Qin, Daoliang Zhang

**Affiliations:** ^1^Shanghai Chest Hospital, Shanghai Jiao Tong University, Shanghai, China; ^2^Danbury Hospital, Danbury, Connecticut, USA

## Abstract

Extrapulmonary vein focal sources have been recognized as the source of atrial fibrillation in some cases, and empiric electric isolation of the left atrial appendage has been proposed for long-standing persistent atrial fibrillation by some. Here, we present a case of redo ablation of long-standing persistent atrial fibrillation in which the left atrial appendage played a key role in maintaining AF during ablation, and atrial fibrillation was terminated by electrical isolation of the LAA. During the ablation, a rare phenomenon of half of the atria in atrial fibrillation while the other half of the atria in atrial flutter was seen.

## 1. Introduction

The role of focal sources in the initiation and maintenance of atrial fibrillation (AF) have been well recognized, with pulmonary veins (PV) being the most common location of these foci [1]. PV isolation (PVI) is now the cornerstone of catheter ablation for AF [2]. However, PV isolation alone cannot maintain sinus rhythm in many patients, especially in patients with long-standing persistent AF (LSPAF) [3]. So far, extra-PV focal sources maintaining AF have been reported in the superior and inferior vena cava [4, 5], coronary sinus [6], ligament of Marshall [7], crista terminalis, and posterior left atrium [8]. Takashi and colleagues have reported a case of paroxysmal AF originating from a focus localized to the left atrial appendage (LAA) [9]. Here, we present a case of redo ablation of LSPAF in which the LAA played a key role during ablation and AF was terminated by electrical isolation of the LAA.

## 2. Case Report

A 61-year-old male with LSPAF was referred to our center for repeat catheter ablation. He had previously undergone catheter ablation for persistent AF 12 years ago during which PVI, linear ablations (LA roof line and mitral isthmus line), and complex fractionated atrial electrogram (CFAE) ablations were done. He was in sinus rhythm on follow-up at 3 months and 1 year but was then lost to follow-up. He was again in AF 2 years ago and was referred again to our center for redo ablation.

**Figure 1:**
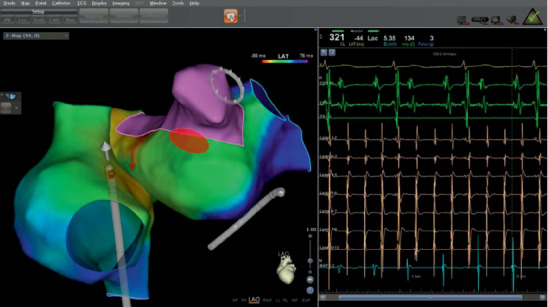
Activation mapping of the LA and RA. The LAA and surrounding area is in AF (pink, average cycle length 150 ms); the rest of the atria including the anterior and lateral walls of the LA, the interatrial septum, and the RA are in atrial flutter (cycle length 330 ms, earliest in CS 9, 10). Red circle marked the area ablated during CFAE ablation.

**Figure 2:**
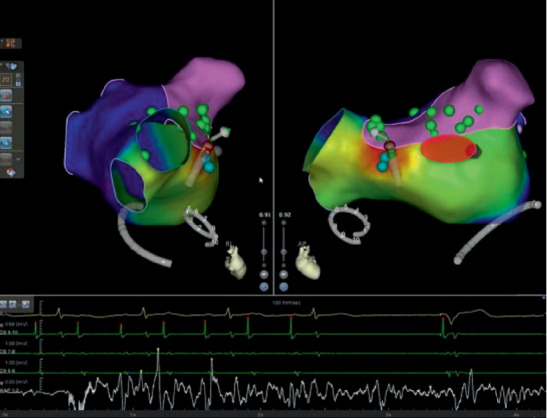
Termination of the atrial flutter. Activation mapping showed the mechanism of the atrial flutter to be focal (atrial activation spanning 145 ms, less than one-half of the cycle length of the atrial flutter, which was 330 ms); the earliest point of activation is on the left side of the interatrial septum. A linear area of double potentials with a gap in the middle can be seen from the base of the LAA to the right PV antrum (green dots). The gap is the area of the earliest activation. Radiofrequency energy delivery at the gap (35 W, 3 seconds) terminated the atrial flutter. A paced signal can also be seen from the temporary pacemaker inserted for protection during the procedure. Red circle marked the area ablated during CFAE ablation.

**Figure 3:**
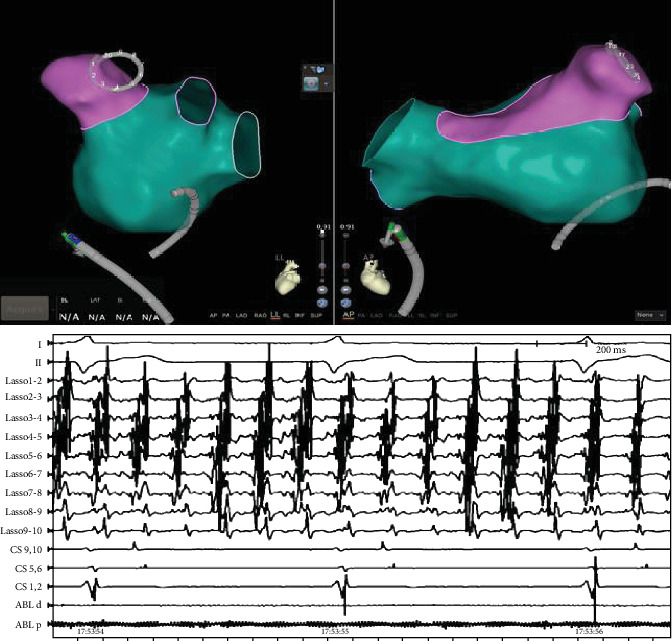
Activation mapping and intracardiac electrograms showing the LAA and surrounding area in AF (pink on activation map, lasso in the LAA) while the rest of the atria (green on the activation map) was in sinus rhythm.

**Figure 4:**
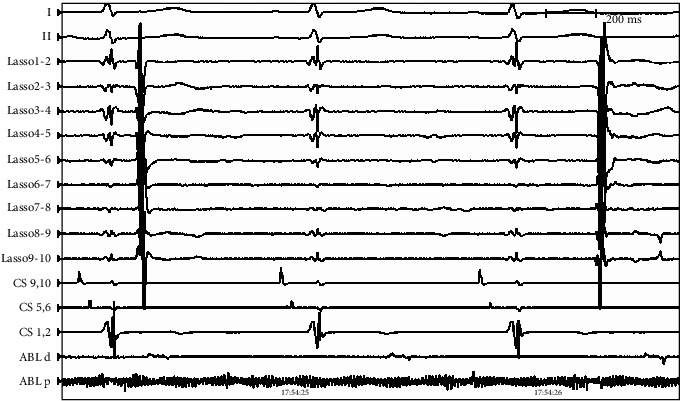
Intracardiac electrograms showing slow spontaneous potentials within the LAA after LAA electrical isolation (lasso in the LAA, no cardioversion done).

During the ablation procedure, reconnection sites were ablated and PVI was again achieved. Gaps in the mitral isthmus line were ablated. During CFAE ablation on the LA anterior wall (red circle in Figures 1 and 2), AF terminated to atrial flutter on the coronary sinus (CS) lead (cycle length 330 ms, earliest in CS 9, 10). Activation mapping of the LA and right atrium (RA) showed atrial flutter on the anterior and posterior walls of the LA as well as the RA (cycle length 330 ms, earliest in CS 9,10), but AF in the LAA and surrounding atrial area (marked as pink in Figure 1, average cycle length 150 ms). Activation mapping showed the mechanism of the atrial flutter to be focal (Figure 2. Atrial activation spanning was 145 ms, less than one-half of the cycle length of the atrial flutter, and earliest on the left side of the atrial septum. A line of double potentials (green dots in Figure 2, from the basal LAA to the right PV antrum) with a gap in the middle can be mapped on the anterior wall of the LA. The gap was the area of earliest activation. Radiofrequency energy (35 W, 3 seconds, saline irrigation 25 ml/min) delivery at the gap area terminated the atrial flutter. On repeat mapping of the LA, sinus rhythm can be seen in most of the LA while the LAA and its surrounding area remained in AF (pink area in Figure 3). As the anterior LA line was being drawn, with the lasso in the LAA, the potential in the LAA can be observed to slow down and then disconnect from the rest of the atrium (Figure 4). After observation of 30 seconds, there was no reconnection of the LAA to the rest of the LA.

The patient was discharged on no antiarrhythmic medication and was anticoagulated with rivaroxaban. On follow-up, he remained in sinus rhythm 9 months after ablation.

## 3. Discussion

To our knowledge, this is one of the first detailed reports of a redo ablation of LSPAF in which the LAA played a key role in maintaining the AF during ablation and AF was terminated with LAA electrical isolation. A rare phenomenon of half of the atrium in AF while the other half was in atrial flutter was also seen during the procedure.

PVI is the well-recognized cornerstone for AF catheter ablation, but pulmonary vein isolation alone is often insufficient to maintain sinus rhythm, especially in patients with LSPAF. Various additional ablations including linear and CFAE ablations have not consistently been shown to be superior in clinical trials.

Takashi and colleagues first reported a case of paroxysmal AF treated successfully with LAA isolation in 2005. Di Biase and colleagues then reported in 2010 that LAA firing can be seen in as much as 27% of patients presenting for repeat ablation of AF, and LAA electrical isolation can decrease AF recurrence in these patients [10]. Later in the BELIEF trial, Di Biase and colleagues reported that in patients with LSPAF, empirical electrical isolation of the LAA for both initial ablation, and redo procedure improved long-term freedom from atrial arrhythmias without increasing complications [11].

Though no increase in thrombotic events were seen in the BELIEF study, there are concerns for increased thrombus formation in an electrically isolated LAA and some have reported a higher rate of stroke or TIA after LAA electric isolation [12]. Therefore, long-term anticoagulation is very important if LAA isolation was done during the ablation. Another possible way to mitigate the risk of thrombus formation in an electrically isolated LAA is LAA occlusion. Indeed, the WATCH-Rhythm trial is ongoing to investigate combining AF ablation with LAA electric isolation and LAA closure with a WATCHMAN device in a single procedure.

Other concerns about LAA electric isolation include technical difficulties due to the relative thick tissues at the base of LAA which may cause reconnection after isolation and the thin tissues in some areas of LAA which may make accidental perforation more likely [13, 14].

Our case showed that LAA isolation can be essential for selecting patient presenting for redo ablation of LSPAF. In the era of individualized AF ablation, we feel it is reasonable to do routine careful mapping of the LAA during ablation for LSPAF and isolate the LAA if mapping indicates that LAA is involved in the maintenance of AF.
